# Identifying intragenic functional modules of genomic variations associated with cancer phenotypes by learning representation of association networks

**DOI:** 10.1186/s12920-022-01298-6

**Published:** 2022-07-06

**Authors:** Minsu Kim, Jennifer E. Huffman, Amy Justice, Ian Goethert, Greeshma Agasthya, VA Million Veteran Program, Yan Sun, Rachel McArdle, Louis Dellitalia, Brady Stephens, Kelly Cho, Saiju Pyarajan, Kristin Mattocks, John Harley, Jeffrey Whittle, Roy Mathew, Jean Beckham, River Smith, John Wells., Salvador Gutierrez, Kimberly Hammer, Pran Iruvanti, Zuhair Ballas, Stephen Mastorides, Jonathan Moorman, Saib Gappy, Jon Klein, Nora Ratcliffe, Ana Palacio, Olaoluwa Okusaga, Maureen Murdoch, Peruvemba Sriram, Dean P. Argyres, Todd Connor, Gerardo Villareal, Scott Kinlay, Shing Shing Yeh, Darshana Jhala, Neeraj Tandon, Kyong-Mi Chang, Samuel Aguayo, David Cohen, Satish Sharma, Mark Hamner, Suthat Liangpunsakul, Michael Godschalk, Kris Ann Oursler, Mary Whooley, Jennifer Greco, Sunil Ahuja, Joseph Constans, Paul Meyer, Michael Rauchman, Richard Servatius, Rachel Ramoni, Sumitra Muralidhar, J. Michael Gaziano, Melinda Gaddy, Agnes Wallbom, James Norton, Timothy Morgan, Todd Stapley, Peter Liang, Sujata Bhushan, Frank Jacono, Daryl Fujii, Philip Tsao, Donald E. Humphries, Grant Huang, James Breeling, Jennifer Moser, Jessica V. Brewer, Juan P. Casas, Kelly Cho, Lori Churby, Luis E. Selva, Mary T. Brophy, Nhan Do, Philip S. Tsao, Shahpoor Alex Shayan, Stacey B. Whitbourne, Patrick Strollo, Edward Boyko, Jessica Walsh, Saiju Pyarajan, Elizabeth Hauser, Scott L. DuVall, Samir Gupta, Mostaqul Huq, Joseph Fayad, Adriana Hung, Junzhe Xu, Kathrina Alexander, Robin Hurley, Jack Lichy, Hongyu Zhao, Peter Wilson, Brooks Robey, Prakash Balasubramanian, Ioana Danciu

**Affiliations:** 1 Computer Science and Mathematics Division, Oak Ridge National Laboratory, Oak Ridge, TN USA; 2 Center for Population Genomics, MAVERIC, VA Boston Healthcare System, Jamaica Plain, MA USA; 3 Massachusetts Veterans Epidemiology Research and Information Center, Veterans Affairs Boston Healthcare System, Boston, MA USA; 4 Department of Veterans Affairs Connecticut Healthcare System, West Haven, CT USA; 5 Yale School of Medicine, New Haven, CT USA; 6 Information Technology Services Division, Oak Ridge National Laboratory, Oak Ridge, TN USA; 7 Computational Sciences and Engineering Division, Oak Ridge National Laboratory, Oak Ridge, TN USA; 8 Advanced Computing for Health Sciences Group, Oak Ridge National Laboratory, Oak Ridge, TN USA; 9 Department of Biomedical Informatics, Vanderbilt University, Nashville, TN USA

**Keywords:** Genome-wide Association Study, Network Representation Learning, Machine Learning

## Abstract

****Background**:**

Genome-wide Association Studies (GWAS) aims to uncover the link between genomic variation and phenotype. They have been actively applied in cancer biology to investigate associations between variations and cancer phenotypes, such as susceptibility to certain types of cancer and predisposed responsiveness to specific treatments. Since GWAS primarily focuses on finding associations between individual genomic variations and cancer phenotypes, there are limitations in understanding the mechanisms by which cancer phenotypes are cooperatively affected by more than one genomic variation.

****Results**:**

This paper proposes a network representation learning approach to learn associations among genomic variations using a prostate cancer cohort. The learned associations are encoded into representations that can be used to identify functional modules of genomic variations within genes associated with early- and late-onset prostate cancer. The proposed method was applied to a prostate cancer cohort provided by the Veterans Administration’s Million Veteran Program to identify candidates for functional modules associated with early-onset prostate cancer. The cohort included 33,159 prostate cancer patients, 3181 early-onset patients, and 29,978 late-onset patients. The reproducibility of the proposed approach clearly showed that the proposed approach can improve the model performance in terms of robustness.

****Conclusions**:**

To our knowledge, this is the first attempt to use a network representation learning approach to learn associations among genomic variations within genes. Associations learned in this way can lead to an understanding of the underlying mechanisms of how genomic variations cooperatively affect each cancer phenotype. This method can reveal unknown knowledge in the field of cancer biology and can be utilized to design more advanced cancer-targeted therapies.

## Introduction

Genome-wide Association Studies (GWAS) correlate specific genomic variations with phenotypes. They are being actively applied in cancer biology to investigate the link between an individual’s genomic variations and cancer phenotypes, including susceptibility to certain types of cancer or predisposed responsiveness to treatments. GWAS has identified many common genomic variants associated with cancer phenotypes. A recent review study by Sud et al. [1] highlighted hundreds of loci associated with increased cancer risk in a variety of cancer tissues, including breast, prostate, lung, colorectal, pancreatic, gastric, renal, and bladder cancers.

Cancer phenotypes, such as early-onset and late-onset, can be understood as a result of the accumulation of abnormal gene functions, where each gene function can be compromised by genomic variants within each gene [2]. The implications of individual genomic variants on gene function have been actively investigated using genomic data. Substantial amounts of genomic variants have been identified with the potential to affect gene function in a variety of mechanisms, including premature stop, splice site, frameshift insertion and deletion (InDel), missense, untranslated region (UTR), promoter, proximal enhancer, protein binding sites, and RNA binding sites [3].

A collection of genomic variants within each gene can cooperatively alter the expression level of a gene [4] and the structure of its gene product [5]. This implies that cancer phenotypes can be cooperatively influenced by two or more genomic variations within genes that interact with each other [2]. The widespread linkage disequilibrium (LD) of the human genome also suggests that there may be unknown functional associations among individual genomic variations within genes [6]. To understand how genomic variations cooperatively affect specific cancer phenotypes, it is required to first identify associations among them.

There are some challenges in identifying associations among genomic variants within each gene. First, there are no metrics available that can encompass both the co-occurrence between variations and the correlation between the co-occurrence and a given cancer phenotype. This makes it difficult to generate machine learning models that require quantifiable features that well represent associations among genomic variants within each gene. Second, there is no effective and unbiased way to learn associations from pairwise relationships between variations. A computational framework capable of extracting usable representations from data with a graph structure is required, where each node is each variant, and the edge is the degree of association between the two variants. Lastly, there is no systematic way to test how robust these learned associations are.

Biological networks have been actively used to learn associations between biological entities, such as protein-protein interaction (PPI) networks and gene co-expression networks. Many studies have used these network approaches to capture biologically meaningful subgroups or gene subsystems in which genes interact with each other [7–9]. In this study, we consider the individual genomic variations within each gene as entities and extend the biological networks approach to learn associations between them from data.

One of the most effective ways to extract information from a network is to use a network representation learning technique such as DeepWalk [10] and GloVe [11]. DeepWalk is a graph embedding algorithm that uses a deep learning architecture. It learns vector representations from a given graph structure, where the representation encodes relationships between nodes [10]. It first generates a document-like input by sampling the nodes with random walks, and then uses natural language processing (NLP) techniques on the input data to generate vector representations. The learned representation space reflects the relative distance between nodes. GloVe takes the input structure as a global matrix and applies matrix factorization to get vector representations [11]. DeepWalk relies on local contexts to learn representation while GloVe uses global statistics. Both approaches are known to have good performances in NLP tasks such as word analogy inference [10, 11].

**Fig. 1:**
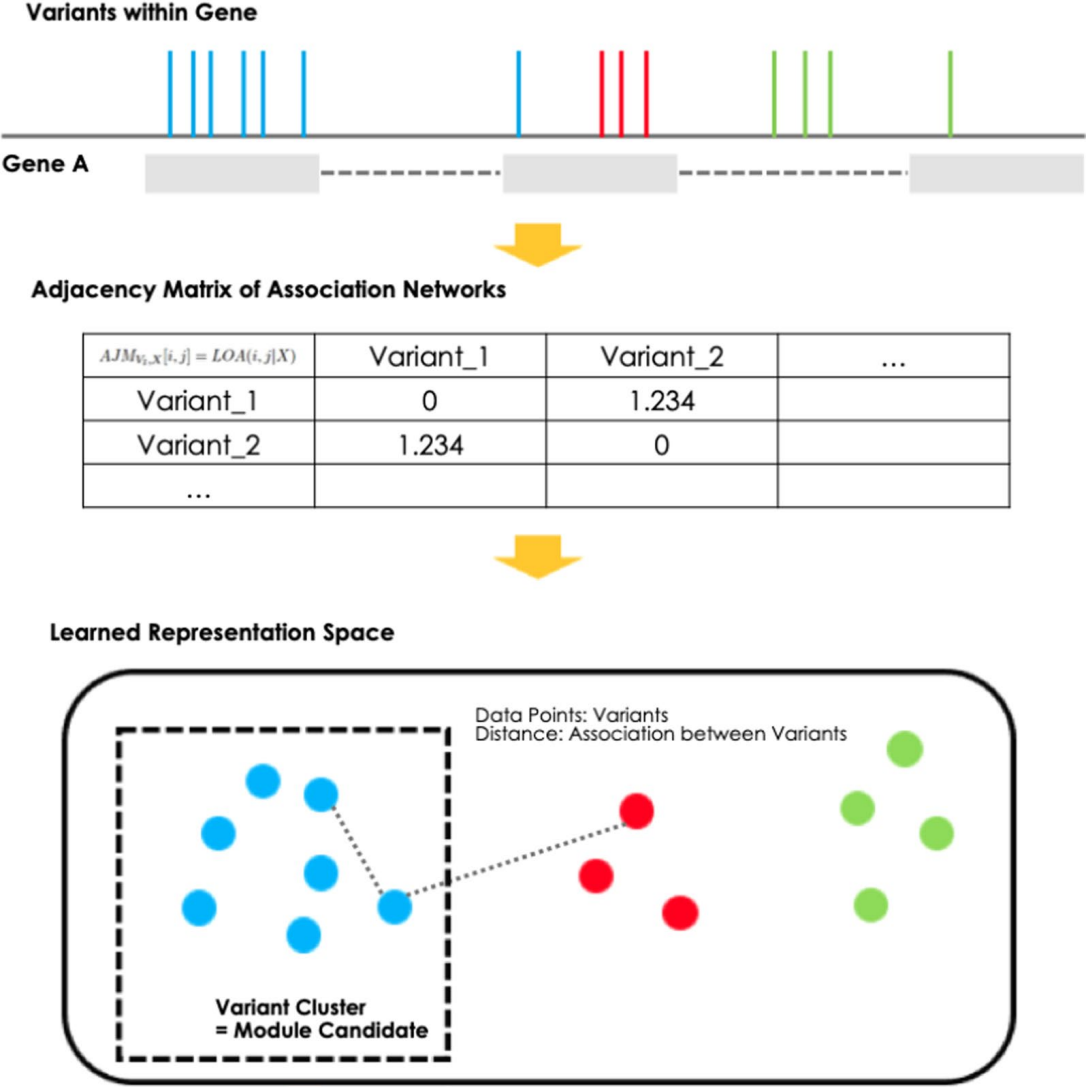
Identification of functional module candidates within a gene. First, the proposed method generates an association network by calculating the LOA between each variant within a gene. It then learns the associations from the network to produce a representation that can be used to identify module candidates

We devised a computational framework for identifying associations among variants by learning network representations. Here, we define the association between two variants by two criteria: (1) the two variants must co-occur frequently, and (2) this co-occurrence must be correlated with a given cancer phenotype. We devised a metric that can quantify both criteria at once to define the level of association (LOA) between two variants for a given cancer phenotype. By measuring the LOA for every pair of variants within a gene, it is possible to construct an association network where each node is each variant and the edge weights are the LOAs between them (Fig. 1). It is possible to infer modules of variants in a data-driven manner using representations learned from the constructed network.

Prostate Cancer (PC) is the leading cancer diagnosis in U.S. men with an estimated 191,930 new cases expected in 2020 and the second most common cause of cancer deaths in U.S. men, accounting for about 33,330 deaths in 2020 [12]. PC is known to be a heterogeneous disease that includes both a chronic phenotype of old age and an aggressive phenotype such as clinically advanced early-onset PC. High-throughput sequencing technology offers opportunities to elucidate biomarkers at the molecular level [13]. However, the clinical and molecular characteristics of early-onset PC still have not been well described [14]. This creates an opportunity for large-scale machine learning genomics approaches to contribute to elucidating the mechanisms of early-onset PC at the molecular level.

We applied the proposed approach to the prostate cancer cohort provided by the Veterans Administration’s (VA) Million Veteran Program (MVP) to identify intragenic functional modules associated with early-onset prostate cancer. Our cohort had 33,159 PC patients (3,181 early-onset and 29,978 late-onset). Note that the scope of the study included only early and late-onset patients with prostate cancer and that the methodology was also evaluated using only the prostate cancer cohort.

The following sections describe (1) how to define associations between genomic variations for a given cancer phenotype, (2) how to learn representations from defined association networks between variations, and (3) how to identify sets of genomic variations within each gene as candidates for functional modules associated with a given cancer phenotype and (4) how to evaluate the proposed method.

## Materials and methods

Our study used DNA samples and phenotypic data from Million Veterans Project (MVP). The MVP program recruited individuals aged 18 to >100 years old from 63 Veterans Affairs Medical Centers across the United States. We used MVP release 19.2 for this analysis [15]. This approach was applied to a cohort with 33,159 prostate cancer patients (3,181 early-onset and 29,978 late-onset). We identified 2,146,891 genomic variations within 5,298 genes. Note that variants here include only single nucleotide variations (SNVs) and InDels.

Our project was approved by the VA Central IRB. All participants enrolled in the MVP have signed an Informed Consent document allowing the use of their data by approved researchers in accordance with the MVP data access policy.

### Terminology

- A genomic variation, variation, genomic variant, or variant refers to individual SNVs and InDels at specific genomic locations.
- Association refers to the co-occurrence between two genomic variations or a correlation between a specific variation and a phenotype.
- An intragenic functional module or module within a gene refers to a set of genomic variants located within each gene, where members co-affect the structure or function of the gene product.
- A module candidate refers to a set of variants within a gene that is inferred from the data as potential candidates for a module.
- A phenotype associated module refers to a module candidate identified as having a statistically significant association with a given phenotype.

### Defining associations between genomic variations

Identifying genomic variations that are associated with each other starts with defining what is association. There are two different aspects of associations to be considered in this context: (1) Co-occurrence between two genomic variations and (2) Correlations between the co-occurrence and a given cancer phenotype. We addressed this by defining the level of association between two variations *A* and *B* for a given phenotype *X* as the following Eqs. (1a, 1b).

1a $$\begin{aligned} LOA(A,B|X) = log\left( \frac{JCS(A,B|X=1)}{JCS(A,B|X=0)}\right) , \end{aligned}$$

1b $$\begin{aligned} JCS(A,B|X=k) = \frac{P(A\cap B|X=k)}{P(A\cup B|X=k)} , \end{aligned}$$

*LOA*(*A*, *B*|*X*) represents the level of association between the two variations *A* and *B* for a given phenotype *X*. $JCS(A,B|X=k)$ is the Jaccard similarity [16] of the two variations *A* and *B* when *X* is *k*. Where *k* is 0 or 1. Where $X=1$ represents the sample with the given phenotype *X*, and $X=0$ is vice versa. $P(A\cap B|X=k)$ represents the intersection of samples with phenotype values of *k* and both *A* and *B* variations, whereas $P(A\cup B|X=k)$ indicates the union. Hence, *JCS* is the ratio of intersection to the union, which is how frequently the two variations co-occur. *LOA* represents the log ratio of *JCS* for samples with a given phenotype to samples without the phenotype. Thus, a positive *LOA* indicates a positive association between two variations for a given phenotype, a negative *LOA* indicates the opposite, and a value of 0 indicates no association. By defining a metric in this way, it is possible to measure how differentially two variants co-occur in samples with different phenotypes.

### Constructing association networks of intragenic variations

After annotation, each genomic variation is assigned to the gene in which it is located. We then computed the *LOA* between all pairwise variations (Eq. 1a) to construct a graph structure represented by the adjacency matrix *AJM* (Eq. 2). Each node in the constructed graph or association network is a genomic variation and the edge weight represents the level of association between the two variations.

2 $$\begin{aligned} AJM_{V_l,X}[i,j] = LOA(i,j|X) , \end{aligned}$$

For *i* and $j \in V_l$, where $V_l$ is the set of all genomic variations identified in gene *l*. The edge weight between two nodes or variations *i* and *j* for a given phenotype *X* can be defined as *LOA*(*i*, *j*|*X*), which is also the value of the adjacency matrix $AJM_{V_l,X}[i,j]$. Since the metric is symmetric, $AJM_{V_l,X}[i,j]$ equals $AJM_{V_l,X}[j,i]$.

### Representation learning for the defined association networks

The constructed association network contains valuable information about how each genomic variation in each gene is associated with each other. There are two major approaches to learning associations between entities, (1) Local context window approaches such as DeepWalk [10], (2) Global matrix factorization approaches such as GloVe [11]. In the task of learning associations between genomic variations, there was no systematic comparison of which approach worked better, so we implemented both methods to suit our problem and compared the results. Hereafter, the local context window approach will be referred to as the LCW approach and the global matrix factorization approach will be referred to as the GMF approach.

**Fig. 2:**
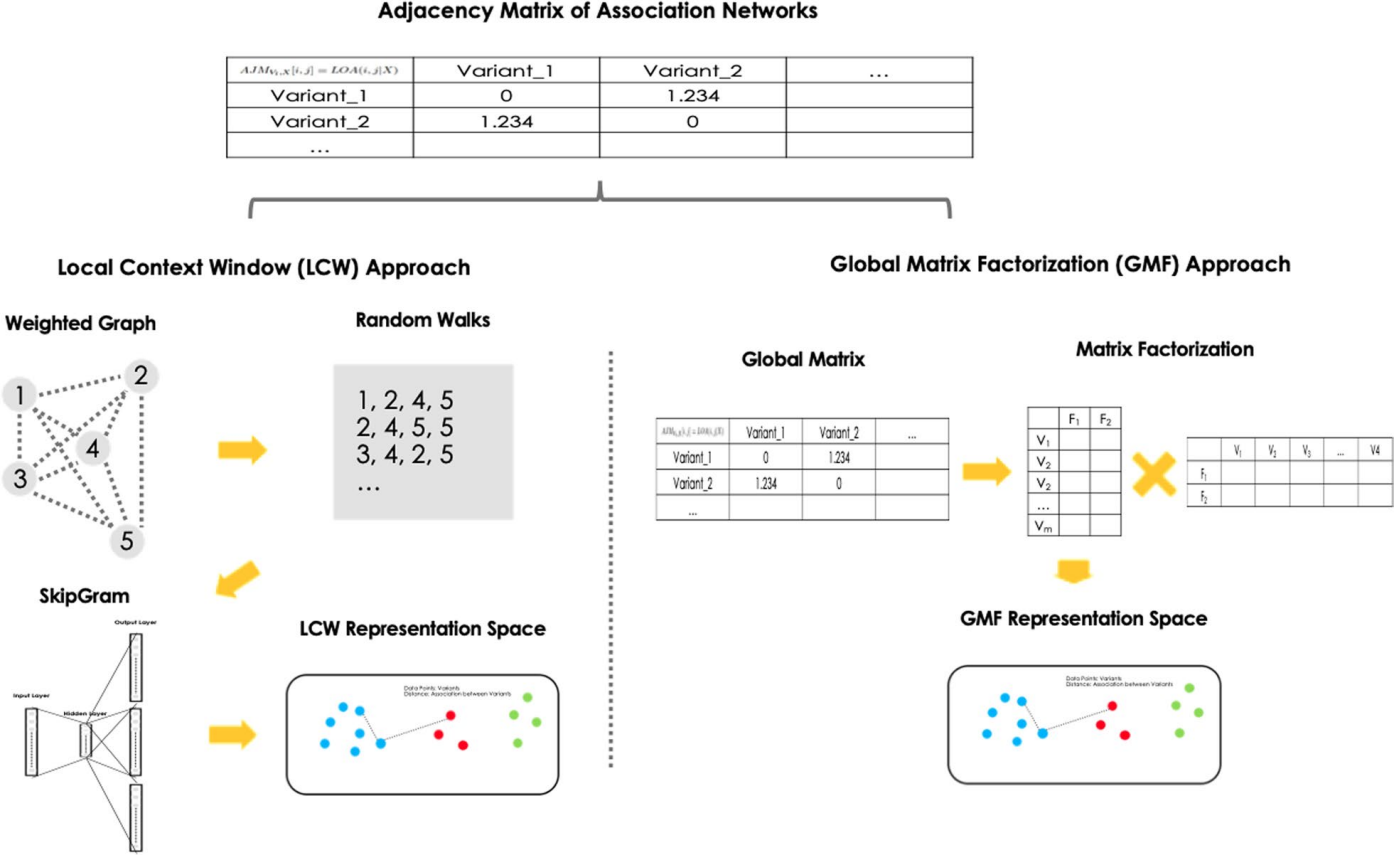
Description of sub-approaches to learning representations of an association network. The first sub-approach is to use SkipGram, the other is to use global matrix factorization

Both approaches have a similar input/output structure that takes a pairwise adjacency matrix $AJM_{V_l,X}[i,j]$ (Eq. 2) and then learns a vector representation for each variant (Fig. 2). LCW takes a matrix as a weighted graph where each node is a variant. Then it samples a weighted random walk starting at each node as a corpus of words (Fig. 2). This approach uses Word2Vec (i.e. SkipGram) [17] to learn the representation of each variant in the corpus by processing it as a word in the document. GMF takes the matrix then applies matrix factorization to produce a vector representation (Fig. 2). It extracts a representation by decomposing a given adjacency matrix into two low-dimensional rectangular matrices. Principal component analysis (PCA) [18] and non-negative matrix factorization (NMF) [19] were used for the decomposition.

### Identifying module candidates using the learned representations

After learning the representation space from the data, we applied a general data mining technique, hierarchical clustering [20] to extract variant clusters that can be considered module candidates. The optimal number of clusters *k* was determined using the silhouette score [21] (Fig. 1). For each gene, the optimal number of variant clusters was found by choosing the *k* that maximizes the silhouette score. Then each cluster was considered a module candidate.

### Measuring the association between module candidates and cancer phenotypes

To assess the significance of each module candidate, we established a metric that measures how strongly the module candidate is associated with a given phenotype. The metric computes the correlation between each patient’s module status and phenotype. Here, we defined the status of each module for each patient as either activated or inactivated. For a given threshold *thr* between 0 and 1, if the module activation level *MAL* of patient *p* for module *m* exceeds *thr*, then the module activation status *MAS* of patient *p* for module *m* is set to activated (Eqs. 3a, 3b, 3c).

3a $$\begin{aligned} MAL(p, m) = \frac{|R_m \cap F_p|}{|R_m|} , \end{aligned}$$

3b $$\begin{aligned} MAS(p, m) = {\left\{ \begin{array}{ll} 1, &{} \text {if} \ MAL(p, m) > thr(m) \\ 0, &{} \text {otherwise} \end{array}\right. } \end{aligned}$$

3c $$\begin{aligned} thr(m) = \frac{\sum _{p \in L}{MAL(p,m)}}{|L|} , \end{aligned}$$

*MAL*(*p*, *m*) represents the module activation level of patient *p* for module *m*, where $R_m$ is the set of variants in module *m* and $F_p$ is the set of variants that are found in patient *p*. *MAS*(*p*, *m*) represents the module activation status of patient *p* for module *m* for a given threshold value for module *m*, *thr*(*m*), where 1 indicates activated and 0 indicates the opposite. *the*(*m*) is computed as the mean *MAL*(*p*, *m*) for all patients, where *L* is the set of all patients in the data.

For the confusion matrix described in Table 1, we defined $FEP(m,V_l)$ as the one-sided *p* value of Fisher’s exact test [22] and $FER(m,V_l)$ Defined as the prior odds ratio (Eq. 4). Here, $L_a$ is the set of patients with *MAL* activated and early onset phenotype. $L_b$ is the set of patients with *MAL* activated and late-onset phenotypes. $L_c$ is the set of patients with *MAL* inactivated and early onset phenotype. $L_d$ is the set of patients with *MAL* inactivated and late-onset phenotype. Note that $FEP(m, V_l)$ is defined as a positive one-sided Fisher’s exact test, meaning that it only measures the significance of the positive correlation between module activation and the early onset phenotype.

4 $$\begin{aligned} FER(m, V_l) = \frac{|L_a|/|L_b|}{|L_c|/|L_d|} , \end{aligned}$$

**Table 1:** Confusion matrix for calculating positive one-sided Fisher’s exact test

	Early-onset	Late-onset
Module activated	$L_a$	$L_b$
Module inactivated	$L_c$	$L_d$

$FER(m, V_l)$ is the prior odds ratio of Fisher’s exact test between module activation status and the early-onset prostate cancer phenotype for module m. A higher $FER(m, V_l)$ value indicates a greater positive correlation between activation of the module *m* and the given phenotype $V_l$, which is the early-onset phenotype in this case, and a lower $FEP(m, V_l)$ indicates a greater positive correlation.

### Evaluation of the approach

**Fig. 3:**
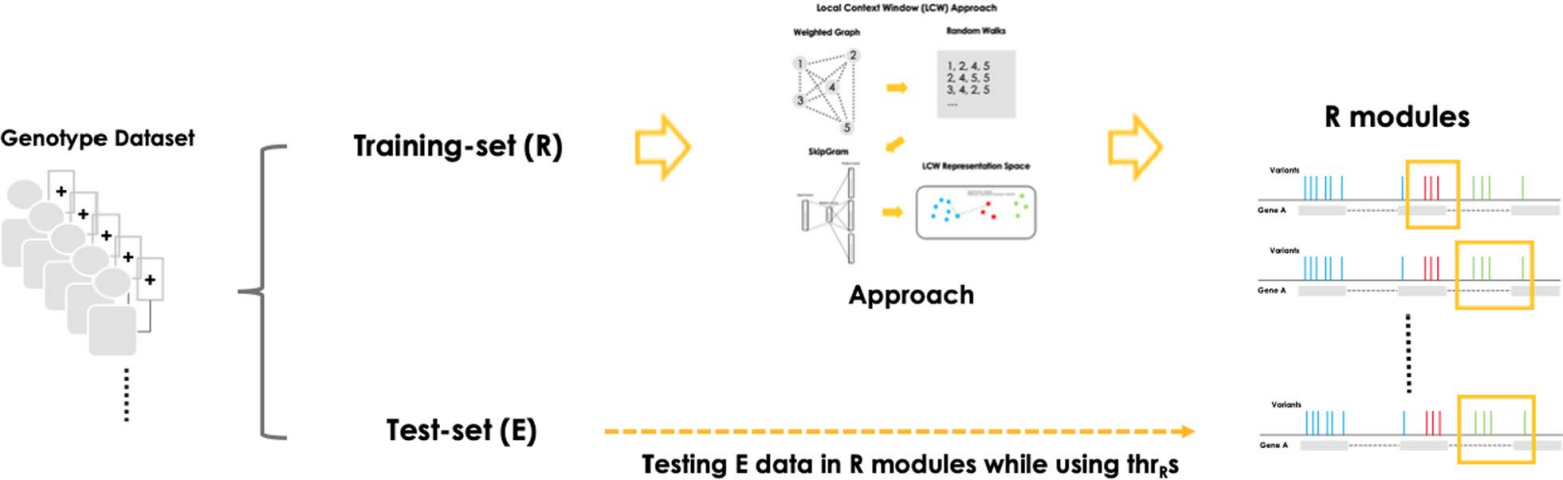
Description of the evaluation scheme. It aims to evaluate the robustness of each sub-approach by measuring reproducibility

One of the main challenges in analyzing genomics data with machine learning approaches is overfitting. It occurs when the model memorizes the training data and the results are not reproduced in other datasets. We measured the robustness of our approach as the model’s ability to handle noise and prevent overfitting, which is measurable through the level of reproducibility of the results between two separate datasets. We divided the data set into training and test datasets to test reproducibility. Here, the training-set contains 16,579 prostate cancer samples (1590 early-onset, 14,989 late-onset) and the test-set contains 16,580 samples (1591 early-onset, 14,989 late-onset) (Fig. 3).

Three sub-approaches with different implementations were prepared for evaluation: (1) LCW approach using SkipGram (LCW-SG), (2) GMF approach using PCA (GMF-PCA), and 3) GMF approach using NMF (GMF-NMF). In addition, two alternative approaches were prepared as controls: (1) the individual variant approach (CTRL-INV) and (2) the gene-level aggregation approach (CTRL-GLA). CTRL-INV is a control approach that evaluates the performance of individual variants instead of modules, which can show whether it is useful to infer associations between variants. CTRL-GLA is a control approach in which all variants within each gene are considered a single module, which can show whether it is useful to infer modules from the data. A total of five approaches were evaluated in terms of robustness (Fig. 3).

The evaluation process is performed for each sub-approach as follows (Fig. 3). (1) identify module candidates using each sub-approach, (2) measure *FEP* with training data for each module candidate, (3) identify phenotype associated modules with a *p* value threshold of 0.05, (4) measure *FEP* with test data for each phenotype associate module, and (5) calculate the reproduction rate by applying a *p* value threshold of 0.05. For example, if the LCW-SG approach identifies 1000 module candidates from the training data, 100 of them are found to have *FEP*s greater than 0.05. And if nine of them are found to have *FEP*s greater than 0.05 in the test data, then the reproduction rate is 9%. Since the test data were not used to define module candidates, the reproduction rate reflects the expected reproducibility when using the approach on one dataset and validating it on another dataset.

### Implementation of the sub-approaches

As stated, LCW-SG, GMF-PCA, and GMF-NMF are the network representation learning approaches we aim to evaluate, while CTRL-INV and CTRL-GLA are the control approaches that provide a baseline for evaluation.

CTRL-INV is an approach that measures the reproduction rate of individual variants. It calculates the *p* value of the Fisher-exact test to measure the correlation between the early onset phenotype and each variant instead of a module candidate. It does not use any algorithms, models, or parameters. Thus, the reproduction rate here reflects the innate bias between the training and test datasets, meaning that this is a target that an ideal algorithm can achieve. CTRL-GLA is an approach that considers all variants within a gene to constitute a single module, thus it represents a passive algorithm that does not actively identify modules among variants. It can be used to quantify the usefulness of approaches using active algorithms such as LCW-SG, GMF-PCA, and GMF-NMF compared to a passive algorithm.

LCW-SG, GMF-PCA, and GMF-NMF approaches use network representation learning approaches to identify module candidates from given data (Fig. 2). The aforementioned approaches take *AJM* as input, learn vector representations for each variant, and then identify module candidates using hierarchical clustering [20] in the learned representation space, where the optimal number of clusters is determined using the silhouette score [21]. Their difference lies in how the network representation is learned from *AJM*.

In the case of LCW-SG, it starts at each node (i.e. each variant) and extracts 10 weighted random walks of length 10 each to learn the network representation. For example, if a gene has 100 variants, *AJM* represents a graph of 100 nodes, where the edge weights between nodes A and B represent the *LOA* between variants A and B. Since the edge weight reflects the level of association between two variants (Eq. 1a), the weighted random walk contains association information between variants. Therefore, by applying the SkipGram algorithm to the extracted random walk, associations between variants can be encoded into a vector representation.

GMF-PCA learns the vector representation by applying PCA [18] to *AJM* via two-dimensional reduction, whereas GMF-NMF uses NMF [19] with the same number of dimensions. Note that the matrix *AJNM* (Eq. 5) used in GMF-NMF was a matrix with an exponential value for each value of *AJM*, because NMF requires the matrix to be non-zero.

5 $$\begin{aligned} AJNM_{V_l,X}[i,j] = e^{LOA(i,j|X)} , \end{aligned}$$

## Results

As shown in the Table 2, the three approaches identified 14,000–20,000 module candidates in 5270 genes (variants in 28 genes were missing in the training data). LCW-SG identified 20,045 module candidates, of which 517 were found to be phenotype associated in the training data (acceptance rate 2.58%), of which 59 were found to be phenotype associated in the test data (reproduction rate 11.41%). Next, GMF-NMF identified 14,985 module candidates, of which 1728 were identified as having a phenotype association in the training data (acceptance rate 11.53%), of which 257 were identified as being phenotype associated in the test data (reproduction rate 14.87%). Lastly, GMF-PCA identified 20,369 module candidates, of which 2465 were found to be phenotype associated in the training data (acceptance rate 12.10%), of which 342 were identified as phenotype associated in the test data (reproduction rate 13.87%).

CTRL-GLA identified 5270 module candidates for each gene (Table 2), of which 67 were found to be phenotypes associated in the training data (acceptance rate 1.27%), of which 4 were found to be phenotype associated in the test data (reproduction rate 5.97%). This means that all three approaches, LCW-SG, GMF-NMF, and GMF-PCA, performed better in terms of reproduction rate, with the best approach being GMF-NMF, which is 2.49 times larger than CTRL-GLA. This means that GMF-NMF is 2.49 times more reproducible than a passive algorithm. CTRL-INV identified 1,887,981 individual variants as module candidates, of which 305,106 were identified as having a phenotype association in the training data (acceptance rate 16.16%), of which 188,369 were identified as being a phenotype associated in the test data (reproduction rate 61.74%). As shown in Table 2, the reproduction rate of GMF-NMF was 24.09% of CTRL-INV, which is the target value that an ideal algorithm can achieve. This means that the robustness level of the network representation learning approach is as good as 24.09% of the ideal target value.

**Table 2:** Reproducibility test results with a *p* value threshold of 0.05

Approaches	#_of_module_candidates	p_R_ < 0.05	Ratio	p_E_ < 0.05	Ratio
LCW-SG	20,045	517	0.0258	59	0.1141
GMF-NMF	14,985	1,728	0.1153	257	0.1487
GMF-PCA	20,369	2,465	0.1210	342	0.1387
CTRL-GLA	5,270	67	0.0127	4	0.0597
CTRL-INV	1,887,981	305,106	0.1616	188,369	0.6174

## Discussion

GMF-NMF showed the best performance in terms of reproduction rate among the three approaches based on network representation learning (NRL). All three approaches were found to outperform CTRL-GLA. This means that the NRL approach can improve reproducibility when identifying functional modules within genes, regardless of implementation.

### Limitations of the approach

As can be seen from the comparison with CTRL-INV, the best of the three NRL approaches performed 24.09% compared to the control, implying that substantial overfitting still remains. These limitations can be explained in part by the use of primitive algorithms to identify phenotype associated modules. Since our goal was to measure the effectiveness of the NRL method itself, we did not use techniques to reduce overfitting such as data augmentation [23] and regularization [24] in this evaluation. This means that there is room to improve the performance of the approaches by deploying more sophisticated machine learning approaches such as deep neural network architectures and XGBoost [25] that can leverage the aforementioned techniques.

However, as indicated by the reproduction rate of the CTRL-INV approach, even the non-parameterized approach did not achieve 100% reproducibility. In other words, the data is inherently biased and prone to false discoveries. Since these innate biases reside in the data itself, it cannot be addressed by more sophisticated machine learning approaches or other techniques to avoid overfitting. To address these challenges, there is an alternative to improving machine learning approaches by adopting prior knowledge of the associations and interactions between biological entities. In a recent study by Kim et al. [9], an external source of information such as a PPI network was adopted in addition to the given input data so that the model can learn more generalized and reproducible patterns to avoid overfitting.

Unfortunately, it is not easy to apply the aforementioned idea directly to our problem because there is not enough information about the functional associations among variants. As discussed in the Introduction section, the functional and structural associations among variants within a gene are not yet fully understood. We believe that advances in deep neural networks (DNNs) and artificial intelligence (AI) technologies in genomics studies could be a solution to the problem. To the best of our knowledge, there are no viable methods that can identify associations among variants within a gene in terms of implications for protein function and structure. Therefore, this leaves us with two interesting topics for future research. First, to develop a method to learn functional associations between variants by adopting external information, such as linkage disequilibrium (LD) among variants [26, 27], or a method to learn functional associations directly from genomic sequences using AI techniques. Next, to integrate the learned associations between variants with our network representation learning framework.

### Limitations in the cohort selection

Due to the MVP enrollment protocols, and the overall demographics of the VA population, the data used for this study is biased towards individuals who survive to the date of enrollment in the MVP program. Most of the subjects with both early and later onset prostate cancer enrolled in MVP after their diagnosis of Prostate Cancer (92% of early-onset and 83% of later onset). This suggests the possibility of survival bias in both groups, particularly in the early-onset group. However, our paper focuses on methods development, and the intragenic GWAS approach described in this paper is generalizable to other association studies.

### Novelty of learning associations among genomic variations

Learning associations between biological entities have been widely used in the biomedical field, not only in genomics data [9] but also in other clinical data such as electronic health records [28]. However, to our knowledge, no approach has been found that can actively learn associations among genomic variations to target variables such as early and late-onset phenotypes. Therefore, the proposed approach can be of great benefit for further studies pursuing specific target phenotypes.

### Future works

We identified associations between variations within genes. Since the underlying biological mechanisms for each association have not yet been investigated, it is not easy to present the outcomes as meaningful results. For example, suppose we found an association between a particular variant A and B. To reasonably interpret the results, we should also be able to provide actual evidence to support a functional association between them. It could be a linkage related to protein structure or other regulatory implications it may have, such as transcription factor (TF) and microRNA (miRNA) binding sites. Therefore, to evaluate and annotate learned associations in terms of clinical and biological significance, a more systematic framework is needed to evaluate these implications by integrating heterogeneous data sources such as biological networks and sequences.

## Conclusion

This work proposes a network representation learning (NRL) approach and evaluates its utility in the identification of intragenic functional modules of genomic variation within a gene to facilitate understanding of the link between genomic variation and cancer phenotype. This approach was applied to the MVP prostate cancer cohort, which included 33,159 prostate cancer patients, 3,181 early-onset, and 29,978 late-onset. The NRL approach was evaluated in terms of reproducibility. The GMF-NMF approach showed 2.49 times higher reproduction rate than CTRL-GLA using a passive algorithm, while 24.09% compared to CTRL-INV, indicating that there is still substantial overfitting to improve.

To our knowledge, this is the first attempt to use the NRL approach to identify associations between genomic variations within genes. This study made it clear that the NRL approach can improve model performance in terms of reproducibility. Moreover, it opens up ways to study associations among variants to facilitate the understanding of the underlying biological mechanisms of how variants affect cancer phenotypes, which could lead to more advanced therapeutic targets for anticancer therapies.

## Data Availability

Full GWAS summary statistics can be found in dbGaP (https://www.ncbi.nlm.nih.gov/gap/) under the Million Veteran Program accession phs001672.
